# Ferrocholesteric–ferronematic transitions induced by shear flow and magnetic field

**DOI:** 10.3762/bjnano.8.255

**Published:** 2017-11-30

**Authors:** Dmitriy V Makarov, Alexander A Novikov, Alexander N Zakhlevnykh

**Affiliations:** 1 Physics of Phase Transitions Department, Perm State University, Bukireva St. 15, 614990 Perm, Russia

**Keywords:** ferrocholesteric, helix unwinding, liquid crystals, magnetic field, shear flow

## Abstract

We study the unwinding of the ferrocholesteric helical structure induced by a combined action of a magnetic field and a shear flow. Both influences are able to induce the ferrocholesteric–ferronematic transition independently; however, the differences between the magnetic field orientation and the flow alignment direction lead to a competition between magnetic and hydrodynamic mechanisms of influence on the ferrocholesteric structure. We analyze various orientations of a magnetic field relative to the direction of a shear flow. The pitch of the ferrocholesteric helix is obtained as function of the strength and the orientation angle of the magnetic field, the shear velocity gradient and a reactive parameter. Phase diagrams of ferrocholesteric–ferronematic transition and the pitch of the ferrocholesteric helix as functions of the material and the governing parameters are calculated. We find out that imposing a shear flow leads to a shift of the magnetic field threshold. The value of the critical magnetic field depends on the magnetic field orientation, the velocity gradient, and the viscous coefficients. We show that the interplay of a magnetic field and a shear flow can induce reentrant orientational transitions that are ferrocholesteric–ferronematic–ferrocholesteric.

## Introduction

The dispersing of nanoparticles of different nature (e.g., carbon nanotubes, ferromagnetic or ferroelectric particles, quantum dots, silica particles) into liquid crystals (LCs) leads to a change in the properties of the composite system [1–9]. The physical properties of these kinds of soft condensed matter are much richer than those of the original liquid crystal materials. Depending on the type of impurity particles in liquid-crystalline colloidal systems new mechanisms for the orientation structure control are revealed. One of such materials is a ferroliquid crystal, a highly dispersed magnetic suspension of anisometric particles of a ferro- or ferrimagnet, in which the carrier liquid is a liquid crystal [10]. In contrast to pure liquid crystals, which are diamagnetic media with a quadrupole mechanism of interaction between the LC and an external magnetic field, the addition of magnetic particles into the LC leads to the appearance of an additional dipole mechanism of the influence on the system. Some types of synthesized ferroliquid crystals have ferromagnetic properties and are highly sensitive to an external magnetic field [11–12].

If the carrier medium in the ferroliquid crystal is a cholesteric liquid crystal (CLC), such a composite system is called ferrocholesteric (FC). A distinctive feature of a CLC is the presence of a supramolecular helical structure, which is very sensitive to various external influences (mechanical, electromagnetic, acoustic, and temperature fields). It is known [13] that with a positive anisotropy of the magnetic susceptibility χ*_a_* of a liquid crystal, the director tends to orientate along the magnetic field. The application of a magnetic field perpendicular to the axis of the cholesteric helix causes the director to rotate in the direction of the field and a subsequent unwinding of the spiral structure. The pitch of the helix increases with the increase of the field and becomes infinite (the cholesteric–nematic transition) above a critical field strength *H*_c_ [13]. Because χ*_a_* is small, the value of *H*_c_ in a CLC is relatively large. The inclusion of a small amount of magnetic particles into a CLC, as shown in [14–17], significantly changes the critical field for the unwinding of the ferrocholesteric helix.

Moreover, the flow of a liquid crystal with a velocity gradient leads to its orientation [13]. Thus, the shear flow of a nematic liquid crystal orients its director at an angle called the Leslie angle [13], which is determined by the ratio of the rotational viscosity coefficients of the LC. As shown in [18–19], a shear flow can unwind the spiral structure of the cholesteric liquid crystal. The diagrams of the cholesteric–nematic orientational transitions induced by a shear flow and a magnetic field were calculated in [20]. The deformation of the spiral orientational structure of a cholesteric liquid crystal under a combined effect of a magnetic field and a shear flow was theoretically investigated in [21], where it is shown that the competing effects of a magnetic field and a shear flow lead to the appearance of reentrant nematic–cholesteric–nematic phase transitions under the rotation of the magnetic field in the plane of shear. The influence of a magnetic field on the helical orientational structure of FCs in the absence of shear flow was considered in [14]. The authors studied the unwinding of an FC helix and obtained the ferrocholesteric–ferronematic transition field as a function of the material parameters of a suspension. It was shown that the critical field is strongly reduced in a dipolar regime of spiral unwinding for rigid planar coupling between liquid crystalline and magnetic subsystems. Magnetic field-induced orientational phenomena in an FC with soft homeotropic coupling were discussed in [15–17]. The orientational phases induced by a magnetic field and a shear flow in a ferronematic (i.e., in a nematic liquid crystal doped with magnetic particles) were studied both in an infinite sample [22], and in a restricted geometry [23]. It was revealed that shear flow can lead to the shift of the field thresholds or to a “smoothing” of the magnetic field-induced transitions in ferronematics. In FCs the possibility of controlling the spiral structure and its unwinding by a combined action of a magnetic field and a shear flow has not been studied. This problem is being investigated in the present paper.

## Basic Equations

**Figure 1:**
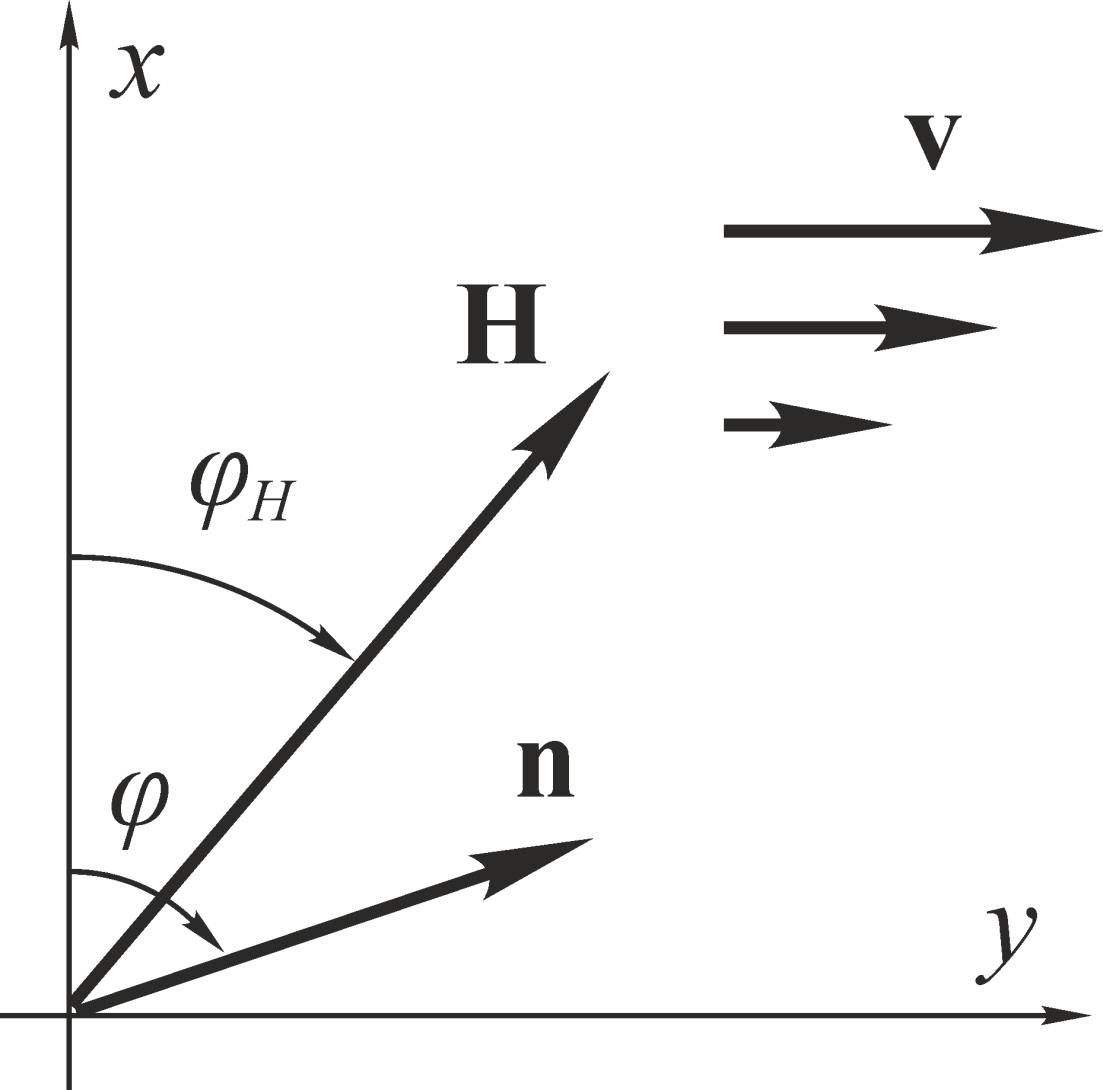
Orientation of the director and the magnetization of a ferrocholesteric in a magnetic field and a shear flow. The *z*-axis is pointed away from the observer.

Let us consider a shear flow with the velocity **v** = [0,*v*(*x*),0] of a ferrocholesteric liquid crystal with the helical axis oriented along the *z*-axis orthogonally to the shear plane *x*–*y* (Figure 1).

The gradient of the velocity of the shear flow *A* = d*v*(*x*)/d*x* is assumed to be constant over the whole sample. Such a flow leads to the aligning of LC molecules in the shear plane at an angle φ_0_ with respect to the flow direction, which in the coordinate system under consideration is determined by the relation [24]:

[1]
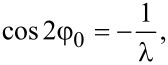


where λ = −γ_2_/γ_1_ is the reactive parameter (λ *>* 0 in an LC composed of rod-shaped molecules [13]), γ_1_ and γ_2_ are the coefficients of the rotational viscosity of an LC. Liquid crystals with λ ≥ 1 are called flow-aligning LCs. If 0 *<* λ *<* 1 the orientation of the director **n** in the flow will not settle to a constant value but to continue to move randomly. The LCs are then called non-flow-aligning LCs.

The coupling conditions between the needle-like magnetic particles and the LC matrix will be considered rigid and planar, so that the director and magnetization will be described by one vector **n**. Due to the helicoidal structure of the director of the CLC matrix, the magnetization vector is also spirally twisted in space, and in this respect the ferrocholesteric is a liquid crystal analogue of a helicoidal ferromagnet. We apply the magnetic field **H** = *H*(cosφ*_H_*, sinφ*_H_*, 0) orthogonally to the axis of the ferrocholesteric helix at an angle φ*_H_* in the shear plane *x*–*y*. We assume the anisotropy of the diamagnetic susceptibility χ*_a_* of a liquid crystal to be positive, so the vector **n** tends to orient in the field direction. In this case the magnetic field and the shear flow act competitively on the ferrocholesteric. Each of these influences orients the director of the ferrocholesteric in its direction in the plane *x*–*y*, inducing the unwinding of its spiral orientational structure.

Let us study orientational effects in a ferrocholesteric liquid crystal, assuming that the distribution of *N* magnetic particles in the volume *V* of the suspension is homogeneous, then their volume fraction in the sample is *f*(**r**) = 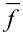 ≡ *Nv*_p_/*V*, where 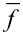 is the average concentration of magnetic particles in the suspension, and *v*_p_ is the volume of a particle.

The equation of motion and the incompressibility condition of a ferrocholesteric liquid crystal in the continuum theory [10,25] can be written as follows:

[2]
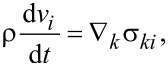


[3]
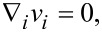


where ρ, *v_i_* and 
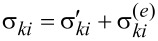
 are, respectively, density, velocity, and stress tensor of a ferrocholesteric liquid crystal; 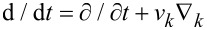 is the total time derivative. Here and below we assume summation over repeated tensor indices.

The viscous stress tensor 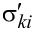, included in the stress tensor σ*_ki_*, has the form

[4]
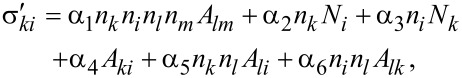


where *n_i_* is the director of the CLC and α*_s_* are the Leslie viscosity coefficients [13] bound by Parodi’s relation α_2_ + α_3_ = α_6_ − α_5_. The vector *N_i_* = d*n_i_*/d*t* − Ω*_ik_n_k_* determines the rate of change of the director *n_i_* relative to the moving medium. The tensors 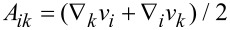 and 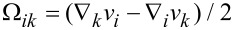 represent the symmetric and antisymmetric parts of the velocity gradient tensor.

The Ericksen stress tensor 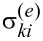, included in the stress tensor σ*_ki_*, is determined by the expression

[5]
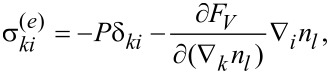


where *P* is the pressure, δ*_ki_* is the Kronecker symbol and *F_V_* is the bulk density of free energy of a ferrocholesteric [10,26]:

[6]
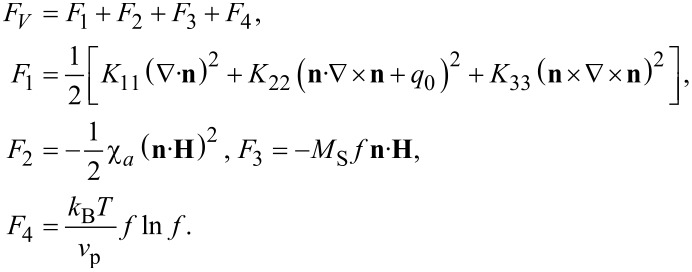


Here *K*_11_, *K*_22_, *K*_33_ are the Frank constants, *q*_0_ is the wave number of the unperturbed spiral structure of a cholesteric liquid crystal (we assume that it is positive), *M*_S_ is the saturation magnetization of the magnetic particles material, *v*_p_ is the volume of a magnetic particle, *f* is the local volume fraction of the particles in suspension, *k*_B_ is the Boltzmann constant and *T* is the temperature.

The contribution of *F*_1_ to the free energy density (Equation 6) determines the energy of orientational elastic deformations of the director field. *F*_2_ is the bulk density of the interaction energy of the magnetic field **H** with an LC matrix (quadrupole mechanism of the magnetic field effect on an FC). *F*_3_ is the bulk density of the interaction energy of the magnetic field **H** with the magnetic moment **μ** = *M*_S_*v*_p_**n** of the particles (the dipole mechanism of the magnetic field effect on an FC). *F*_4_ is the contribution of the entropy of mixing of an ideal solution of magnetic particles to the free energy of a ferrocholesteric. Because of the small volume fraction (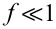) of ferroparticles in the suspension, magnetic dipole–dipole interactions are neglected.

The equation of the director motion has the form [13,27]

[7]
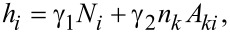


where γ_1_ = α_3_ − α_2_ and γ_2_ = α_3_ + α_2_ are the coefficients of the rotational viscosity of a liquid crystal. The molecular field *h_i_*, acting on the director, is determined by the expression


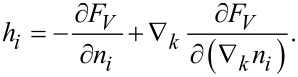


Variation of the free energy is carried out under the additional condition **n**^2^ = 1. Equation 2–Equation 7 determine a complete system of equations for the dynamics of a ferrocholesteric liquid crystal with a rigid planar coupling between the magnetic and liquid crystal subsystems in the absence of segregation.

## Ferrocholesteric in a Shear Flow and a Magnetic Field

For a uniform stationary shear flow with a constant velocity gradient, the incompressibility condition (Equation 3) is fulfilled identically, and the equation of motion of the ferrocholesteric (Equation 2) makes it possible to calculate the pressure. Due to rigid and planar coupling between liquid-crystalline and magnetic subsystems the director and magnetization are described by one vector, which can be written in the following form

[8]
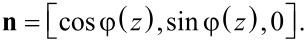


Then the bulk density of the free energy of a ferrocholesteric (Equation 6) has the form

[9]
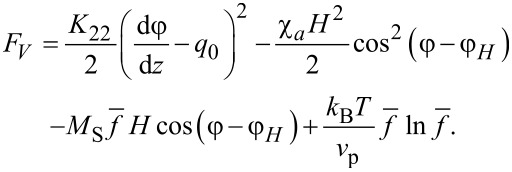


The equation of motion (Equation 7) taking into account Equation 8 yields the following equation for the angle φ(*z*) of the director (and the magnetization) orientation

[10]
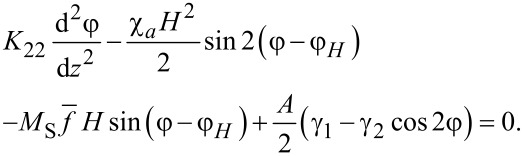


We introduce the following dimensionless quantities:

[11]
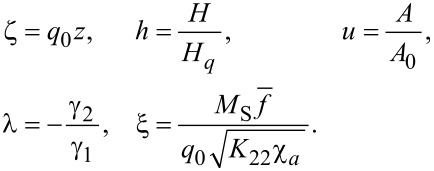


Here, ζ is a dimensionless coordinate and *h* is the dimensionless magnetic field strength. For the unit of the magnetic field, we have chosen the value 
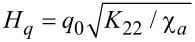
 for which the diamagnetic *F*_2_ and elastic *F*_1_ contributions to the free energy (Equation 6) turn out to be of the same order. It is simply related to the cholesteric–nematic transition field *H_c_* = π*H_q_*/2 [13]. A comparison of the elastic *F*_1_ and ferromagnetic *F*_3_ contributions determines one more typical field 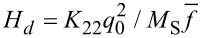, which corresponds to the ferrocholesteric–ferronematic transition field with the predominance of the dipole mechanism of the magnetic field effect over the quadrupole mechanism [14].

The quantity ξ = *H_q_*/*H_d_* is the ratio of two characteristic magnetic fields mentioned above [14]. If ξ *<* 1, i.e., (*H_q_* *< H_d_*), the dominant mechanism of the magnetic field effect on the orientation structure of the ferrocholesteric is the action on the liquid crystal subsystem (quadrupole mechanism). For ξ *>* 1, i.e, (*H_q_* *> H_d_*) the main mechanism is the effect on the impurity magnetic subsystem (dipole mechanism).

The parameter *u* determines the dimensionless value of the gradient of the shear flow velocity with 
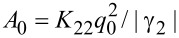
 taken as the unit of measurement. Here, the coefficient of the cholesteric rotational viscosity γ_2_ is taken as an absolute value, since it is negative in LC with rodlike molecules [13]. The reactive parameter λ is the ratio of the coefficients of the liquid crystal rotational viscosity.

We estimate the dimensionless parameters (Equation 11), assuming according to [1,27] that χ*_a_* = 10^−7^, *f* = 10^−5^, *q*_0_ = 10^4^ cm^−1^, *K*_22_ = 10^−7^ dyn, *M*_S_ = 10^2^ G, γ_1_, γ_2_ = 10^−1^ P, *d* = 10^−5^ cm. If we choose *H* = 10^4^ Oe and *A* = 1 s^−1^, we obtain λ ≈ 1, *h* ≈ 1, ξ ≈ 1, and *u* ≈ 10^−2^.

In the dimensionless form, Equation 10 takes the following form:

[12]
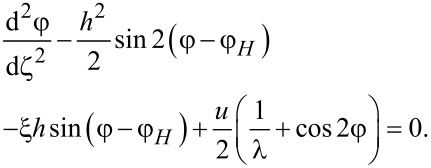


This equation determines the stationary angle φ of the director (and the magnetization) orientation in the ferrocholesteric as a function of the field strength *h* and the orientation angle of the magnetic field φ*_H_*, the parameter ξ of the field effect on the system, the reactive parameter λ, and the velocity gradient *u* of the shear flow. It can also be obtained by using the approach put forward in [18,28–30]. To do this we introduce the effective potential *F*_eff_, which can be written in the dimensionless form as follows:

[13]
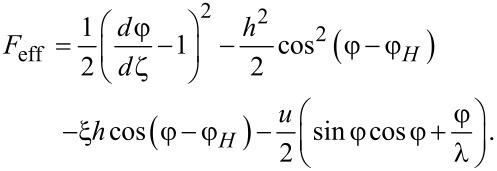


Equation 12 is obtained from the condition of a minimum of this potential: δ*F*_eff_/δφ = 0, where δ/δφ is variational derivative.

## Results and Discussion

Let us analyze the combined effect of the shear flow and the magnetic field on the spiral structure of a ferrocholesteric for various orientations of the magnetic field in the shear plane (Figure 1). In the untwisted (i.e., nematic) phase, in the presence of a magnetic field and a shear flow the director is aligned at the constant angle φ_c_, which can be found from Equation 12:

[14]
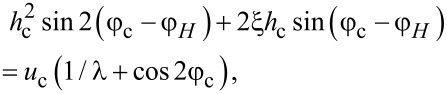


where *h*_c_ and *u*_c_ are the critical values of the field and the velocity gradient at which the ferrocholesteric helix is unwound.

Integrating Equation 12, we obtain

[15]
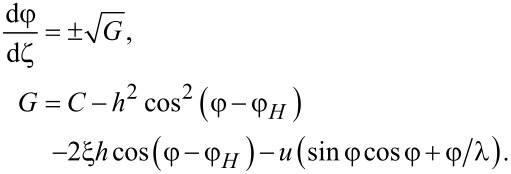


The possible direction of twisting of the ferrocholesteric helix is determined by selecting one of the signs in Equation 15. The unperturbed structure (*h* = *u* = 0) of a ferrocholesteric is described by the solution φ = ζ = *q*_0_*z*. We assume *q*_0_ *>* 0, which corresponds to the upper sign in Equation 15. The integration constant *C* depends on *h*, φ*_H_*, ξ, *u* and λ, and is equal to one in the absence of magnetic field and shear flow.

Let us determine the pitch of the ferrocholesteric helix. Integration over the period of the structure *p* corresponds to a change in the angle φ by 2π, therefore, taking into account Equation 15, we obtain:

[16]
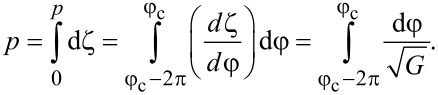


The integration constant *C* can be found from the minimum condition for the effective free energy per turn of the FC spiral structure:


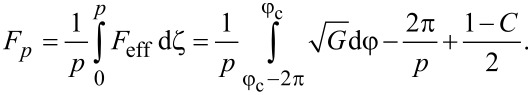


The minimum condition d*F_p_*/d*C* = 0 gives the equation for *C*:

[17]
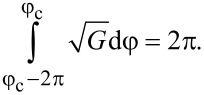


The critical value *C*_c_, corresponding to a ferrocholesteric–ferronematic transition, i.e., the state when the helix pitch diverges, can be obtained from Equation 15. In the untwisted (nematic) phase dφ/dζ = 0, hence


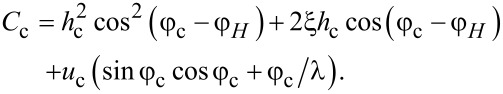


Substituting *C*_c_ in Equation 17, we obtain the condition

[18]
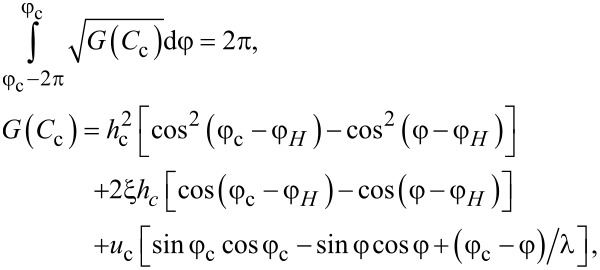


which together with Equation 14 determines the critical values *u*_c_ and *h*_c_, under which the spiral structure of the ferrocholesteric is unwound.

In the absence of magnetic impurities (ξ = 0) and shear flow (*u* = 0) in the untwisted nematic phase, the director is oriented along the magnetic field: φ_c_ = φ*_H_*. In this case, Equation 14–Equation 18, which determine the helix pitch, simplify to:

[19]
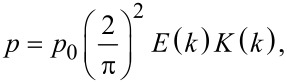


[20]
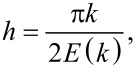


where *p*_0_ = 2π is the pitch of an unperturbed helix of a cholesteric, *K*(*k*) and *E*(*k*) are complete elliptic integrals of the first and second kind [31]. Equation 19 and Equation 20 coincide with the expressions obtained in [32]. At the cholesteric–nematic transition point, the helix pitch (Equation 19) goes to infinity. This pitch corresponds to *k* = 1, for which *K*(1) = ∞ and *E*(1) = 1. Then, Equation 20 implies that the dimensionless critical magnetic field for the cholesteric–nematic transition takes the value *h*_c0_ = π/2.

In the presence of magnetic particles (ξ ≠ 0) without a shear flow (*u* = 0), the director of the ferrocholesteric in an untwisted (i.e., ferronematic) phase directed along the magnetic field at the angle φ*_H_*. We can assign arbitrary values to φ*_H_*, for example, π/2, which corresponds to the orientation of the magnetic field along the *y*-axis. Under this condition the set of Equation 14–Equation 18 takes the form

[21]
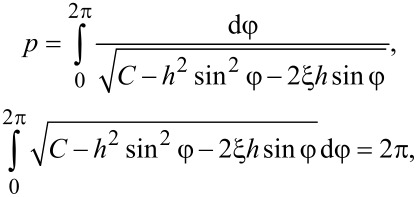


obtained earlier in [14]. The substitution of the critical value 
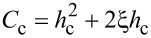
 of the integration constant *C*, at which the helix pitch *p* diverges, in the second equation of the system (Equation 21), gives the dependence of the critical magnetic field strength *h*_c_ on the parameter ξ, which determines the regime of helix unwinding. This dependence can be represented in the parametric form [14]:

[22]
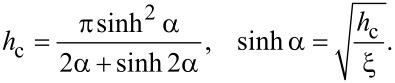


At 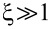 (dipole regime), from Equation 22 we obtain *h*_c_ = π^2^/(16ξ), or in the dimensional form *H*_c_ = π^2^*H_d_*/16. This result coincides with the critical field obtained by Brochard and de Gennes [10]. In another limiting case at 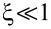 (quadrupole regime) we obtain *h*_c_ = π/2 or in the dimensional units *H*_c_ = π*H_q_*/2. This is a well-known result for pure cholesteric liquid crystals [13].

**Figure 2:**
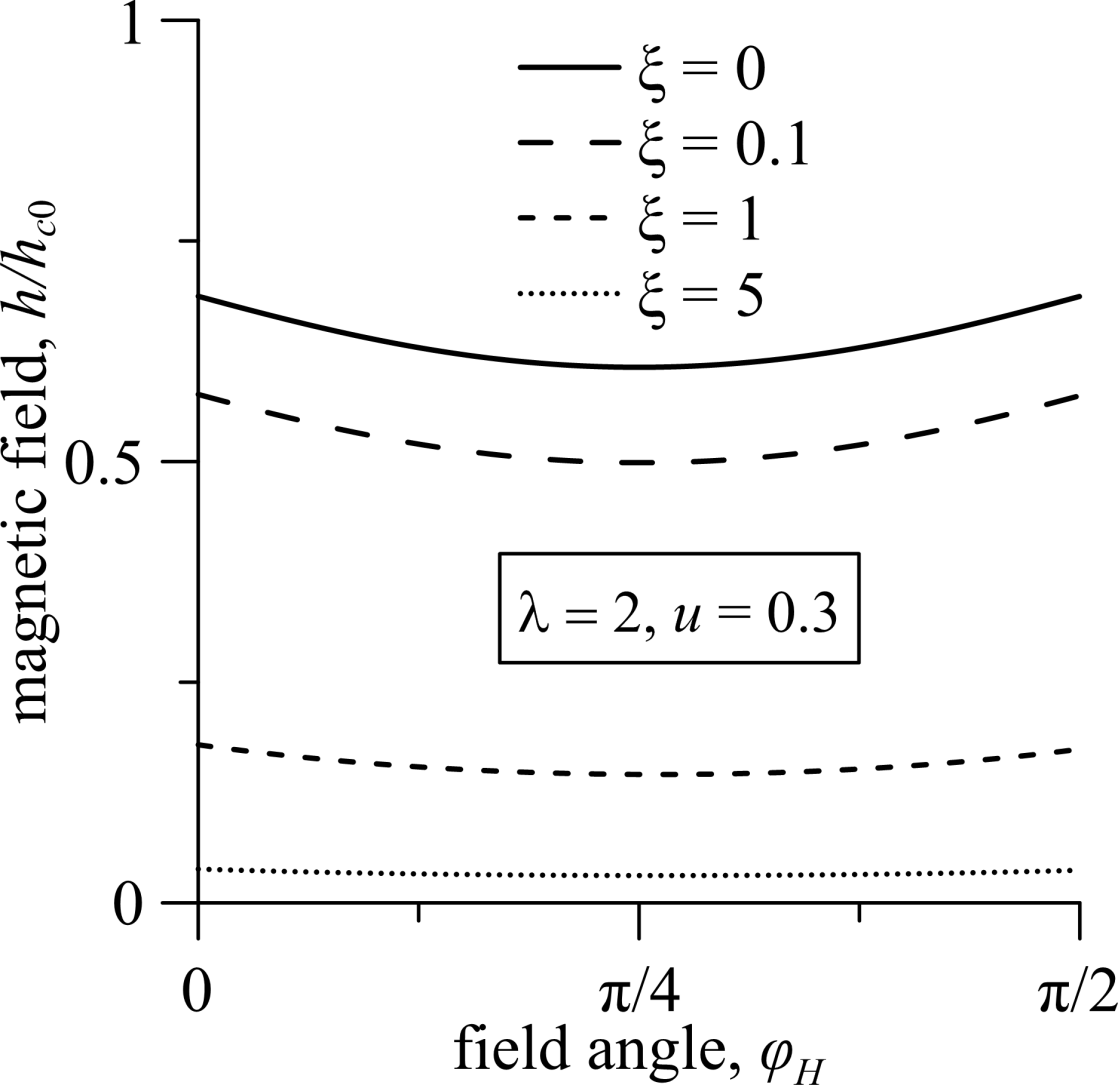
The diagram of the FC–FN phase transition in the plane *h*–φ*_H_* for λ = 2, *u* = 0.3 and different values of the parameter ξ.

Equation 14–Equation 18, which determine the curves of the ferrocholesteric–ferronematic transition caused by a combined action of the magnetic field and shear flow, were solved numerically. The phase diagrams obtained are shown below in Figure 2–Figure 5. The areas that are bounded in these diagrams by the curves and the coordinate axes correspond to the ferrocholesteric phase; the external areas correspond to the ferronematic phase.

Figure 2 represents a diagram of the ferrocholesteric–ferronematic phase transition in the plane *h*–φ*_H_* for different values of the magnetic field effect parameter ξ. It should be noted that the case ξ = 0 corresponds to a CLC without any magnetic impurities. It is seen from the diagram obtained that the critical value of *h*_c_ in an FC for *u* ≠ 0 is smaller than the magnetic field *h*_c0_ required for a cholesteric–nematic transition. Also, for the magnetic field orientation angle 
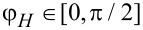
 the transition field reaches the minimum value at φ*_H_* = π/4.

A characteristic feature of the diagram in Figure 2 is the area of ambiguity in which two values of the field orientation angle from the considered interval correspond to the same value of *h*_c_. This means that for the fixed values of *u* and *h* the rotation of the magnetic field can induce a sequence of reentrant ferrocholesteric–ferronematic–ferrocholesteric transitions. The reason for the appearance of reentrant orientational transitions is associated with the competition between the magnetic and hydrodynamic mechanisms of action, which tend to unwind the spiral structure in different directions. As can be seen from Figure 2, the range of values of the magnetic field that enables the reentrant cholesteric phase, decreases with the increase of ξ, i.e., with an increase in the concentration of the magnetic impurity.

**Figure 3:**
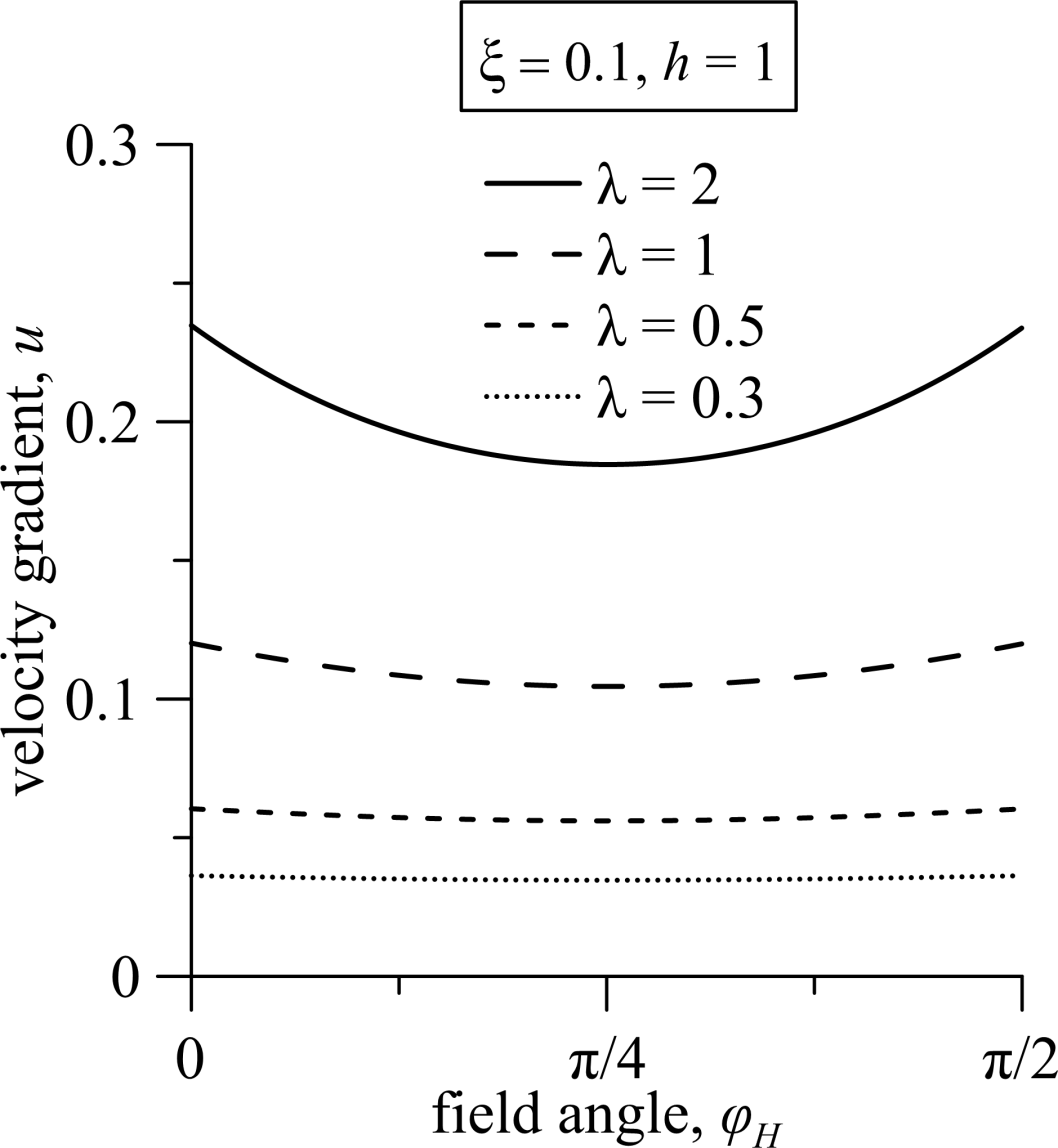
The diagram of the FC–FN phase transition in the plane *u*–φ*_H_* for ξ = 0.1, *h* = 1 and different values of the reactive parameter λ.

Figure 3 represents a diagram of the ferrocholesteric–ferronematic phase transition in the plane *u*–φ*_H_* for *h* = 1 and ξ = 0.1 for different values of the reactive parameter, including the so-called non-flow-aligning LCs (Equation 1) with λ *<* 1. As shown by numerical calculations, the magnetic field stabilizes the orientational structure of an FC in the shear flow, expanding the range of acceptable values of the reactive parameter that have stationary states of the director. This leads to the possibility of unwinding of the FC spiral structure with λ *<* 1. In Figure 3 we see that as λ decreases, the critical value of *u*_c_ decreases and weakly depends on the orientation of the external magnetic field.

**Figure 4:**
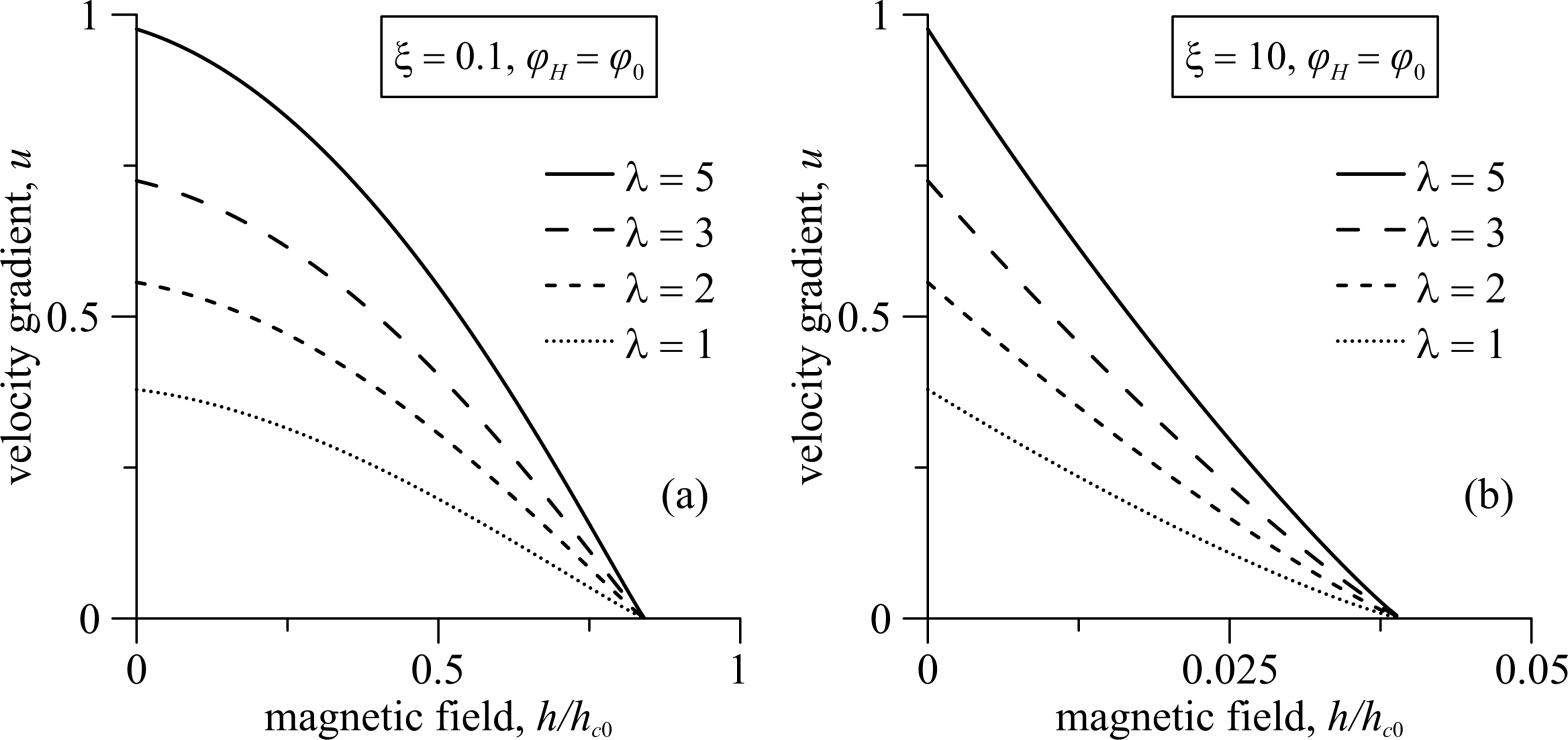
The diagram of the FC–FN phase transition in the plane *u*–*h* for (a) the quadrupole (ξ = 0.1) and (b) dipole (ξ = 10) regimes. In all cases, the magnetic field is oriented at an angle φ*_H_* = φ_0_.

**Figure 5:**
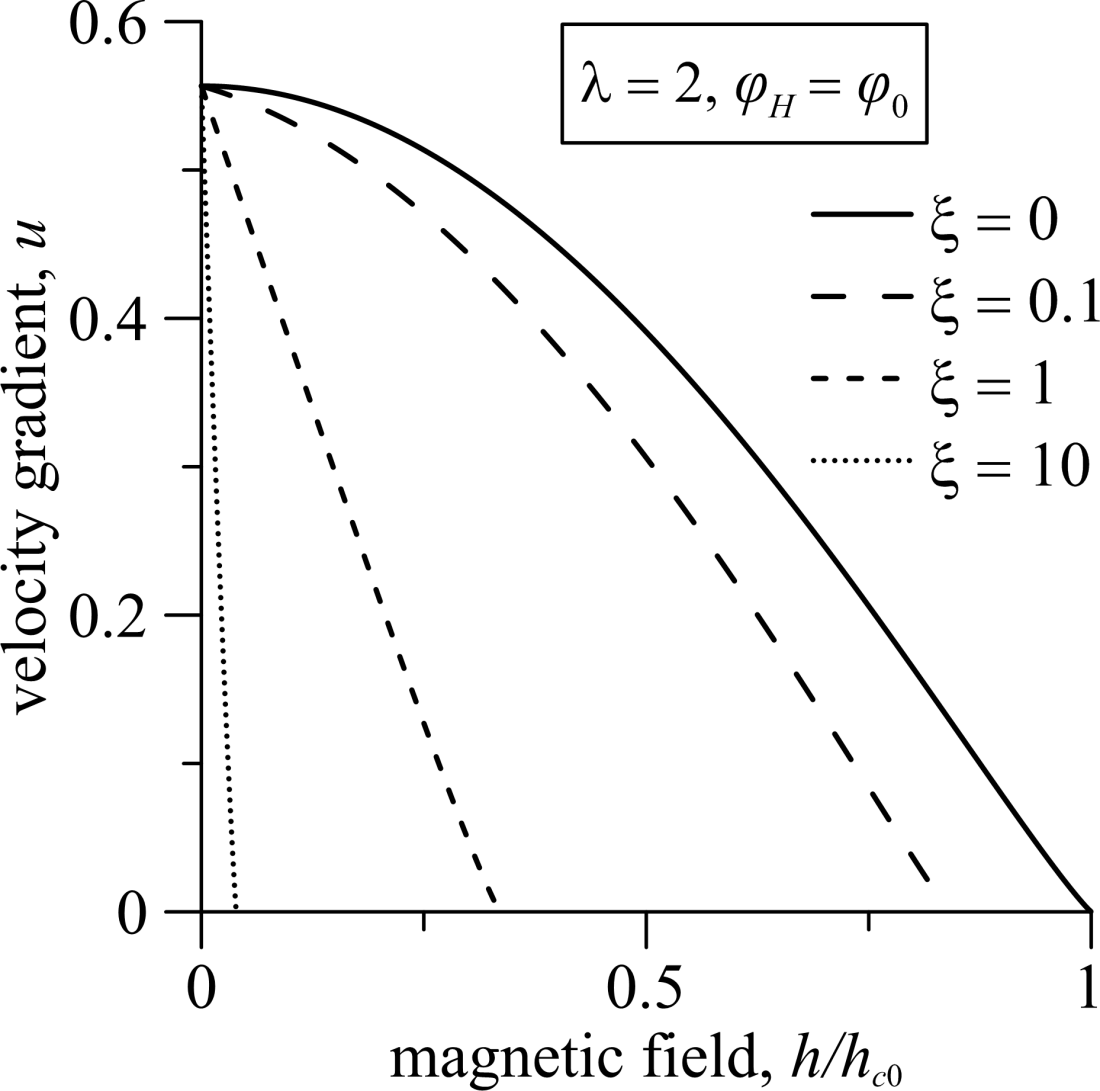
The diagram of the FC–FN phase transition in the plane *u*–*h* for λ = 2, φ*_H_* = φ_0_ and various values of the parameter ξ.

Figure 4 and Figure 5 represent the diagrams of the ferrocholesteric–ferronematic phase transition in the plane *u*–*h* for the magnetic field orientation angle φ*_H_*= φ_0_ for differen values of the reactive parameter λ and the parameter of the magnetic field influence ξ. As seen from Figure 4a,b in the configuration under consideration, the shear flow lowers the critical magnetic field, turning it to zero for *u*_c0_, which depends on the reactive parameter λ. The increase of the reactive parameter leads to an increase in the critical value of the shear velocity gradient *u*_c_. As seen from Figure 5, for a fixed reactive parameter λ and ξ ≠ 0 the transition field *h*_c_ is smaller than the corresponding critical value *h*_c0_ in a pure cholesteric. The effect of the decrease of the transition field occurs more strongly in the dipole regime (ξ *>* 1) with the increase of ξ. Let us keep in mind that the parameter ξ characterizes the intensity of the magnetic field action on the magnetic particles. In the dipole regime the influence of the field on the particles is large compared with its effect on the CLC-matrix, and a sufficiently weak field can unwind the helical structure of FC. In the quadrupole regime (ξ < 1), the field is mainly influenced on the CLC-matrix, and the effects associated with the presence of magnetic impurity are weak. The decrease in the parameter ξ can be interpreted, for example, as a decrease in the magnetic moments of the particles or their concentration. Calculations made for other angles of the field orientation 
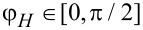
, do not reveal qualitative differences in the diagrams of the FC–FN transitions when comparing them with the dependences shown in Figure 4 and Figure 5.

**Figure 6:**
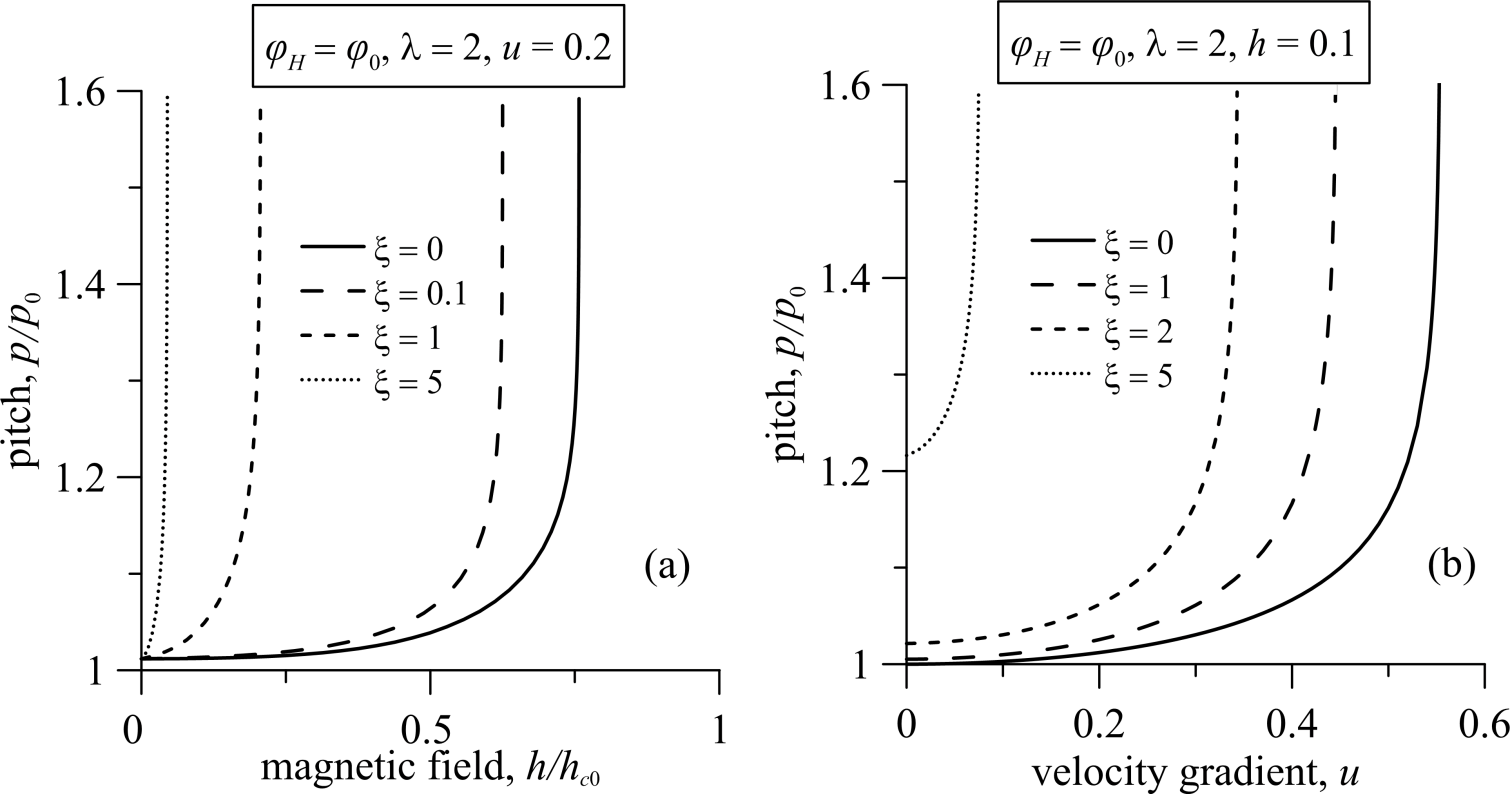
The dependence of the FC helix pitch on (a) the magnetic field strength *h* and (b) the shear flow gradient *u* under different regimes of the magnetic field effect for the angle φ*_H_* = φ_0_; here *p*_0_ = 2π is the pitch of the unperturbed spiral structure.

The pitch of the ferrocholesteric helix is found with the numerical solution of Equation 14–Equation 18. Figure 6 shows the dependence of the reduced pitch *p*/*p*_0_ of the helix on the magnetic field strength *h* and the gradient of the shear flow *u* for different values of the magnetic field influence parameter ξ for the magnetic field orientation angle φ*_H_* = φ_0_.

The pitch of the ferrocholesteric helix, partially unwound by a shear flow (Figure 6a, *u* =0.2) or a field (Figure 6b, *h* = 0.1) grows slowly in weak fields *h* and under small shear gradients *u*. However, it begins to increase strongly when the critical values *h*_c_ or *u*_c_ are approached. Finally, the values of the pitch diverge. As can be seen in the phase diagram in Figure 5, the threshold values *h*_c_ *< h*_c0_ and *u_c_* *< u*_c0_. Here, *h*_c0_ is the critical value of the magnetic field strength of the cholesteric–nematic transition, and *u*_c0_ is the critical magnitude of the velocity gradient in the cholesteric–nematic transition only under shear flow. As can be seen from Figure 6, the increase in the parameter ξ has a much greater effect on the value of the critical field *h*_c_ than on the value of the critical velocity gradient *u*_c_.

**Figure 7:**
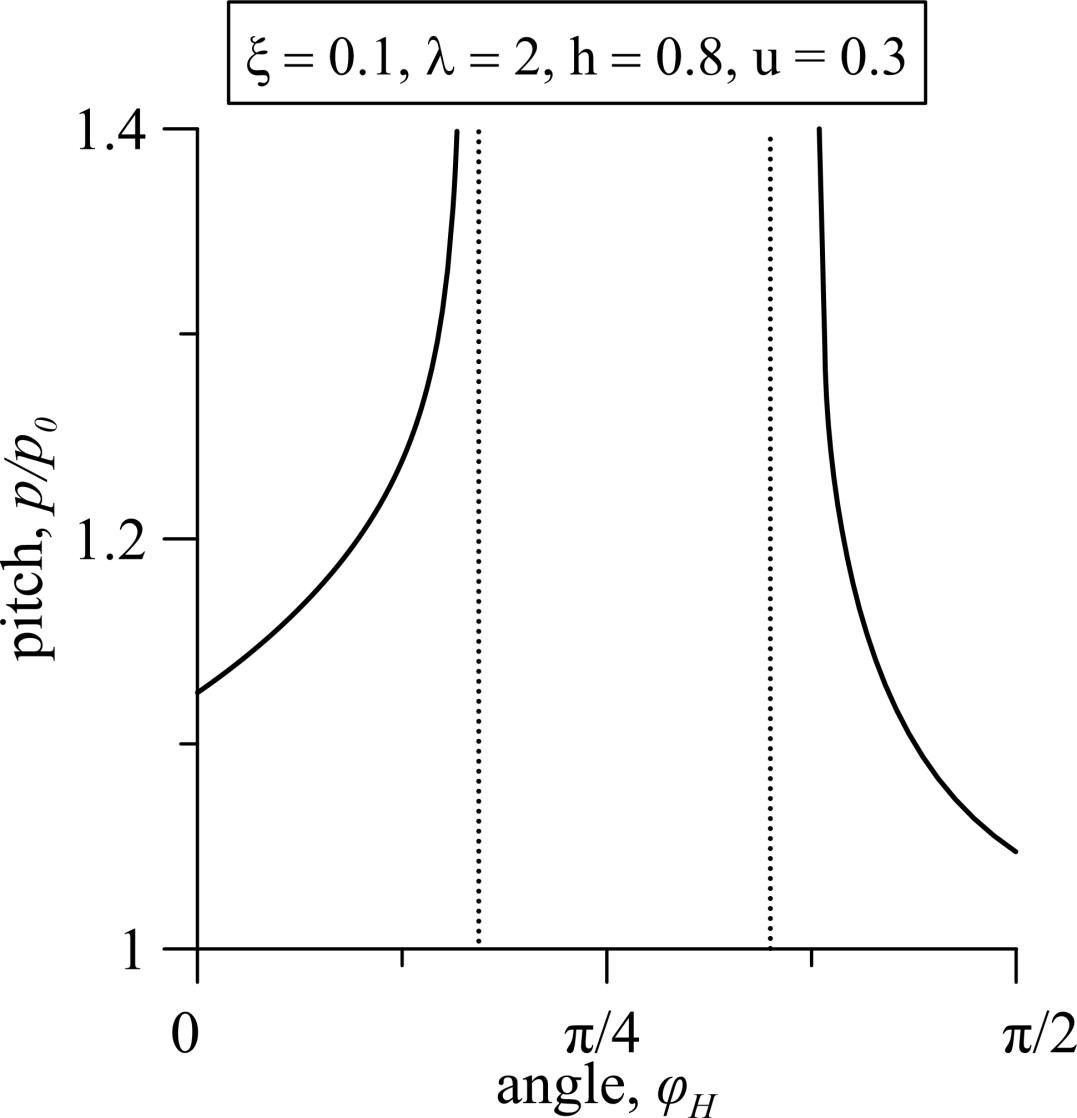
The dependence of the FC helix pitch on the angle of magnetic field orientation φ*_H_* for λ = 2, *h* = 0.8, *u* = 0.3, ξ = 0.1; here *p*_0_ = 2π is the pitch of an unperturbed spiral structure.

Figure 7 shows the dependence of the FC helix pitch on the angle of the magnetic field orientation φ*_H_* for the parameters corresponding to the area of existence of the reentrant orientational transitions (see the curve for ξ = 0.1 in Figure 2). For fixed values of the field *h* and the gradient of the flow velocity *u*, when the ferrocholesteric helix is deformed (*p*/*p*_0_ *>* 1), the turn of the magnetic field corresponding to an increase in the angle φ*_H_*, leads to a more effective influence of a field and a flow on the FC, unwinding the helix and causing the FC–FN orientational transition. The subsequent increase in the angle φ*_H_* decreases the effect of the combined influence of the field and flow on the FC and a reentrant transition to the ferrocholesteric phase occurs. Thus, the reason for the appearance of reentrant orientational phases is due to the competition of hydrodynamic and magnetic influences that align the structure of an FC in different directions in the process of unwinding the helix.

## Conclusion

In this paper the combined effect of a magnetic field and a shear flow on the spiral orientational structure of a ferrocholesteric liquid crystal has been theoretically investigated. The coupling between the LC matrix and the magnetic particles was considered rigid and planar. The axis of the ferrocholesteric helix was oriented orthogonally to the plane of the shear flow with the velocity gradient assumed constant throughout the sample.

We have obtained the diagrams of the ferrocholesteric–ferronematic orientational transitions depending on the shear flow velocity gradient, the reactive parameter, the strength, and the orientation angle of the magnetic field. It has been shown that the magnetic field stabilizes the director orientation in the shear flow and extends the boundaries of the flow alignment area of a CLC with a magnetic admixture. This enables the shear flow to unwind the spiral structure of ferrocholesterics with the reactive parameter λ *<* 1.

We have obtained the dependence of the FC helix pitch of the orientational structure on the magnetic field strength and the gradient of the shear flow velocity in the dipole (ξ *>* 1) and quadrupole (ξ *<* 1) regimes of the magnetic field effect for different values of the reactive parameter.

It has been shown that the addition of ferroparticles to a cholesteric leads to a decrease in the critical magnetic fields of the ferrocholesteric–ferronematic transition. It has been revealed that the competing effect of a magnetic field and a shear flow leads to reentrant orientational transitions (ferrocholesteric–ferronematic–ferrocholesteric), caused by the rotation of the magnetic field in the plane of the shear flow.
